# Leucine supplementation improves leptin sensitivity in high-fat diet fed rats

**DOI:** 10.3402/fnr.v59.27373

**Published:** 2015-06-25

**Authors:** Xue-Wei Yuan, Shu-Fen Han, Jian-Wei Zhang, Jia-Ying Xu, Li-Qiang Qin

**Affiliations:** 1 Department of Nutrition and Food Hygiene, School of Public Health, Soochow University, Suzhou, China; 2 Jiangsu Key Laboratory of Preventive and Translational Medicine for Geriatric Disease, Soochow University, Suzhou, China; 3 Key Laboratory of Radiation Biology, School of Radiation Medicine and Protection, Soochow University, Suzhou, China

**Keywords:** leptin sensitivity, leptin signaling, leucine, high-fat diet

## Abstract

**Background:**

Several studies have reported the favorable effect of leucine supplementation on insulin resistance or insulin sensitivity. However, whether or not leucine supplementation improves leptin sensitivity remains unclear.

**Design:**

Forty-eight male Sprague-Dawley rats were fed with either a high-fat diet (HFD) or HFD supplemented with 1.5, 3.0, and 4.5% leucine for 16 weeks. At the end of the experiment, serum leptin level was measured by ELISA, and leptin receptor (ObR) in the hypothalamus was examined by immunohistochemistry. The protein expressions of ObR and leptin-signaling pathway in adipose tissues were detected by western blot.

**Results:**

No significant differences in body weight and food/energy intake existed among the four groups. Serum leptin levels were significantly lower, and ObR expression in the hypothalamus and adipose tissues was significantly higher in the three leucine groups than in the control group. These phenomena suggested that leptin sensitivity was improved in the leucine groups. Furthermore, the expressions of JAK2 and STAT3 (activated by ObR) were significantly higher, and that of SOCS3 (inhibits leptin signaling) was significantly lower in the three leucine groups than in the control group.

**Conclusions:**

Leucine supplementation improves leptin sensitivity in rats on HFD likely by promoting leptin signaling.

The discovery of leptin has led to astonishing advances in understanding energy homeostasis in rodents and humans (1). Leptin is an adipocyte-secreted hormone that acts on the hypothalamus through a long isoform of leptin receptor (ObR) to control energy balance and body weight. The binding of leptin to ObR activates several signaling pathways, including Janus kinase 2 (JAK2)-signal transducer and activator of transcription 3 (STAT3) (2). By contrast, suppressor of cytokine signaling-3 (SOCS3), an intracellular protein, inhibits leptin signaling (3). In patients with genetic leptin deficiency, exogenous leptin therapy effectively suppresses hyperphagia and corrects metabolic and other abnormalities (4). However, common forms of obesity are typically associated with elevated leptin and resistance to effects of leptin on energy homeostasis (5–7). A combination therapy of leptin and leptin sensitizers may increase leptin sensitivity (7).

Leucine is an essential branched chain amino acid (BCAA). Many studies have demonstrated that leucine plays an important role in regulating energy balance and improving insulin resistance by directly affecting the central nervous system and peripheral tissues (8–10). Some studies have observed a transient effect of leucine on stimulating leptin secretion from adipocytes (11–14), whereas the effects of chronic leucine supplementation on leptin secretion and leptin sensitivity remain unclear (8, 10, 15). In the present study, we primarily investigated whether or not chronic leucine supplementation with high-fat diet (HFD) in rats affects the serum leptin level and ObR expression in the hypothalamus and peripheral adipose tissues.

## Materials and methods

### Animals, diets, and leucine supplementation

Forty-eight 5-week-old male Sprague-Dawley rats (Shanghai Laboratory Animal Center, Shanghai, China) were individually housed in stainless steel wire-mesh cages in an air-conditioned room (22–25°C) with a 12 h light/dark cycle and 55±5% humidity in compliance with the Guide for the Care and Use of Laboratory Animals in Soochow University. After 10 days of acclimatization, the rats were randomly assigned to one of four groups (12 rats per group) fed with HFD (HFD group) or HFD supplemented with different leucine concentrations by weight % (HFD+1.5% Leu group, HFD+3.0% Leu group, and HFD+4.5% Leu group). Leucine was purchased from Shandong Luzhou Amino Acid Co., Ltd. The base diet was modified AIN-93. HFD was made according to feed formation of Teklad Custom Research Diet (TD 06414), and fat sources were lard and soybean oil. HFD contained 5.10 kcal/g with 60.3, 18.4, and 21.3% calories from fat, protein, and carbohydrates, respectively. The animals had free access to food and water. The experiment lasted for 16 weeks.

### Body weight, food intake, and serum leptin level

During the experiment, body weights were recorded weekly. Food was dispensed in a glass container (Natsume Seisakusho Co. Ltd., Tokyo, Japan) and renewed every day. Food intake was measured, and energy intake was calculated once a week. At 16 weeks after the experiment, the rats were deprived of food for 12 h and blood was collected from the femoral artery under ether anesthesia. Serum was separated and stored at −80°C. Peritoneal adipose tissues (except mesenteric one) were quickly removed and weighed. Serum leptin level was determined using commercial ELISA kits (Nanjing Jiancheng Bioengineering Institute, Nanjing, China).

### Immunohistochemistry of hypothalamic ObR

Four animals in each group were perfused transcardially with 4% paraformaldehyde. Brain was carefully removed from the skull, post fixed with the same fixative solution for 24 h, and then dehydrated and embedded in paraffin. Tissue blocks were transversally cut from the head into 3-µm-thick sections and incubated in a blocking solution containing 10% goat serum. The sections were incubated overnight with polyclonal antibodies directed against ObR (1:200; Abcam, Cambridge, MA) and then with biotinylated goat anti-rabbit IgG (diluted 1:500; Sigma) and streptavidin–biotin complex for 1 h each. The sections were washed three times by PBS for 5 min after each incubation. Immunolabeling was revealed with 0.05% diaminobenzidine (Sigma, St. Louis, MO) and 0.03% H_2_O_2_. The sections were counterstained with hematoxylin and then sliced. The slices were observed under a microscope at 40× objective. Five areas of section were randomly selected and quantified as one animal by Image pro-Plus program, and four animals in each group were counted and averaged.

### Western blot of relative protein expression in adipose tissues

Perirenal fat was homogenized in lysis buffer (1:10 wt/vol). Protein concentration was determined using the BCA protein assay. Equal amounts of protein (30–50 µg) were loaded into a 12% sodium dodecyl sulfate –polyacrylamide gel and then transferred to a polyvinylidene difluoride membrane by electrophoretic transfer. After being blocked, the membrane was incubated overnight at 4°C with the following primary antibodies: ObR (1:2,000; Abcam), JAK2 (1:1,000, Cell Signaling Technology, Danvers, MA), STAT3 (1:1,000, Cell Signaling Technology), SOCS3 (1:1,000, Cell Signaling Technology), and β-actin (1:5,000, Millipore, Darmstadt, Germany). After being washed three times, the antigen–antibody complexes were visualized with peroxidase affinipure goat anti-mouse or anti-rabbit IgG antibody (1:2,000) at room temperature for 1 h. Antibody reactivity was detected by chemiluminescence ECL Detection Systems. Blots were performed at least three times to confirm the reproducibility of the results. The intensity of the bands was normalized using each corresponding β-actin density as an internal control. The bands were quantified using Gbox Chemi-XR 5 (Syegene, UK).

### Statistical analysis

Data are reported as means±standard deviation (SD). One-way ANOVA was used to determine differences among groups, followed by the Tukey *post hoc* test. Differences were considered significant at *P*<0.05. All the analyses were performed using SPSS version 17.0 statistical analysis package (SPSS, Inc., Chicago, IL).

## Results

**Fig. 1:**
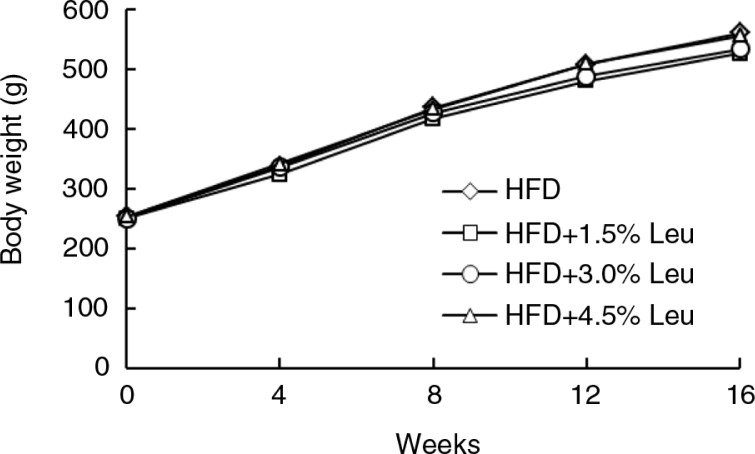
Body weights of rats in the four groups during the experimental period (*n*=12 for each group). HFD, high-food diet; Leu, leucine.

The body weight in the four groups was similar at the baseline and constantly increased over time (Fig. 1). At the end of the experiment, the body weight was slightly low in the HFD+1.5% Leu and HFD+3.0% Leu groups, but no significant difference with the HFD group was observed. Adipose tissue weight was not significantly different among the groups with large variation in group. Despite the slightly low intake in the three leucine groups, no significant differences in food and energy intake existed among the four groups (Table 1).

**Table 1:** Body/adipose weights and food/energy intake with different leucine supplementation at the end of the experiment

	Body weight (g)	Adipose tissue weight (g)	Food intake (g)	Energy intake (kcal)
High-fat diet (HFD)	560.75±45.37	13.70±4.50	19.28±1.28	98.33±6.55
HFD+1.5% leucine (Leu)	528.62±39.96	13.56±4.17	18.52±1.37	94.10±6.96
HFD+3.0% Leu	533.44±33.10	12.93±4.09	18.98±1.11	95.73±5.60
HFD+4.5% Leu	556.08±49.22	13.77±3.40	18.91±1.35	94.64±6.76

Values are means±SD (*n*=12).

**Fig. 2:**
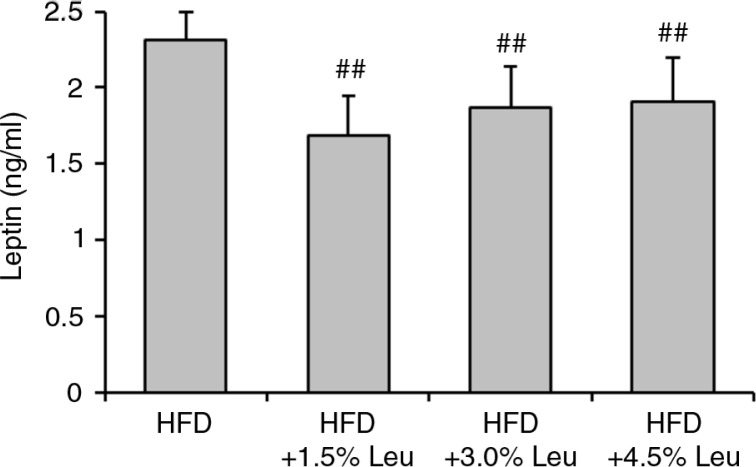
Serum leptin levels in the four groups at the end of the experiment. Values are means for 12 rats with SD represented by vertical bars. The mean value was significantly different from that of the HFD group at ^##^ *P*<0.01. HFD, high-food diet; Leu, leucine.

At the end of the experiment, the serum levels of leptin were 2.31±0.23 ng/mL, 1.69±0.26 ng/mL, 1.87±0.27 ng/mL, and 1.91±0.29 ng/mL in the HFD, HFD+1.5% Leu, HFD+3.0% Leu, and HFD+4.5% Leu groups, respectively. The serum leptin level was significantly lower in the three leucine groups than in the HFD group (all *P*<0.01; Fig. 2). The leptin level in the HFD+1.5% Leu group was the lowest, but there were no significant differences among the three leucine groups.

**Fig. 3:**
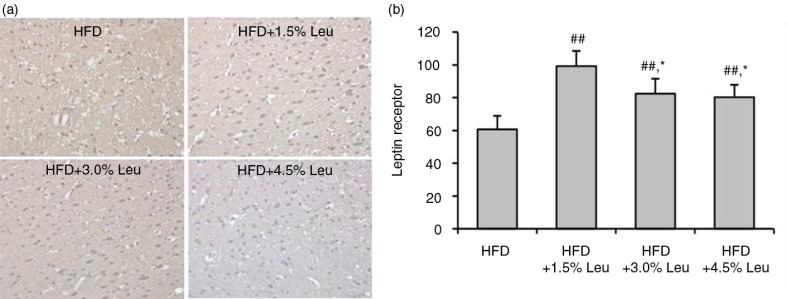
immunohistochemical staining of hypothalamic ObR (a) and comparison (b) in the four groups. (a) Original magnification, 400×. (b) Values are means for four rats with SD represented by vertical bars. The mean value was significantly different from that of the HFD group at ^##^ *P*<0.01 and from that of the HFD+1.5% Leu group at *P<0.05. HFD, high-food diet; Leu, leucine.

Hypothalamic ObR was highly expressed in the rats supplemented with leucine (Fig. 3a). The count of ObR expression was 60.73±8.22, 99.35±9.15, 82.48±9.12, and 80.31±7.49 in the HFD, HFD+1.5% Leu, HFD+3.0% Leu, and HFD+4.5% Leu groups, respectively. The expression was significantly higher in the three leucine groups than in the HFD group (all *P*<0.01). Furthermore, the expression was significantly higher in the HFD+1.5% Leu group than in the HFD+3.0% Leu or HFD+4.5% Leu group (both *P*<0.05) (Fig. 3b).

**Fig. 4:**
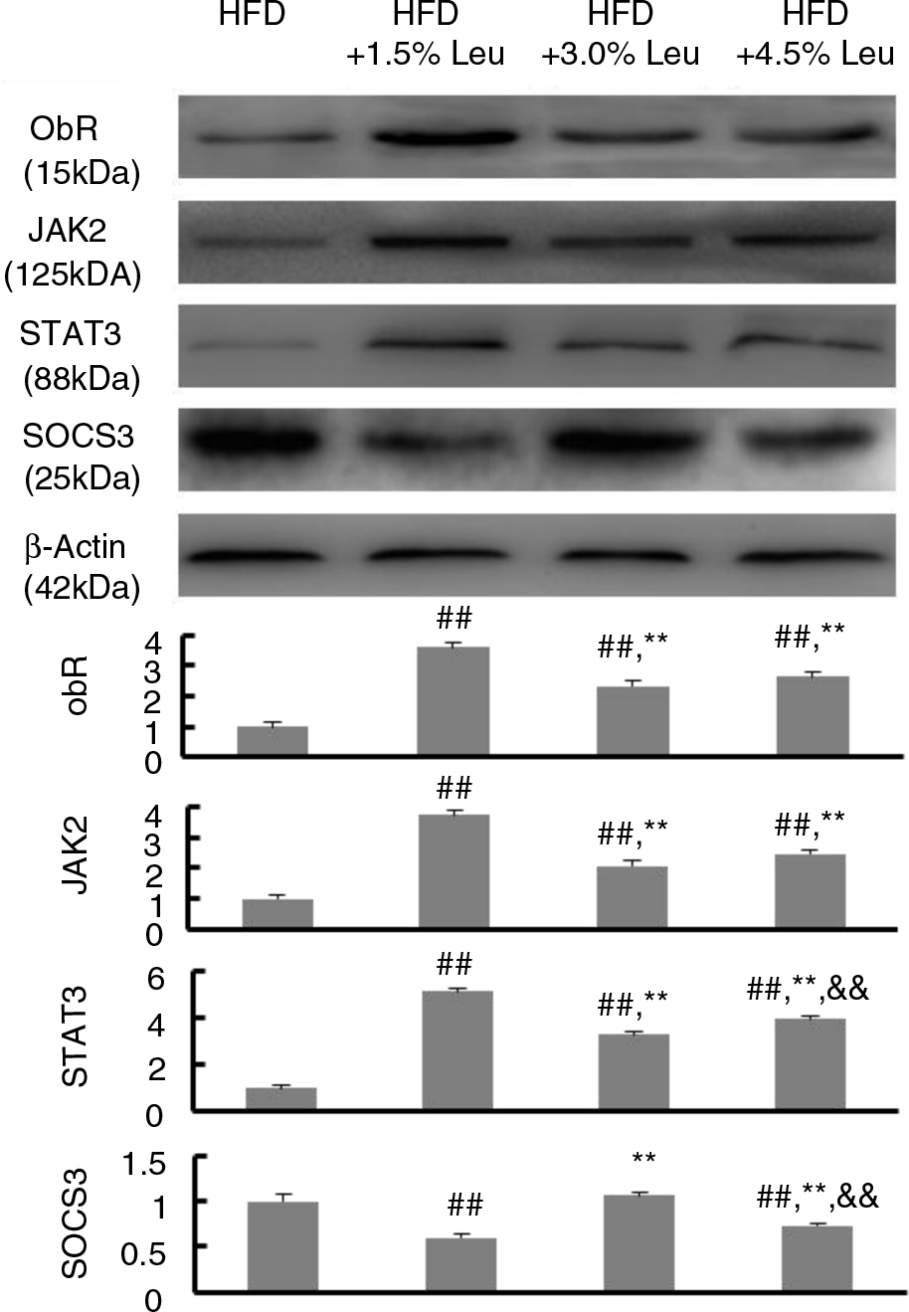
The representative protein expression of ObR, JAK2, STAT3, and SOCS3 in the four groups. Values are means for 12 rats with SD represented by vertical bars. The intensity of the bands was normalized using each corresponding β-actin density. The mean value was significantly different from that of the HFD group at ^##^ *P*<0.01, from that of the HFD+1.5% Leu group at **P<0.01, and from that of the HFD+3.0% Leu group at ^&&^ *P*<0.01. HFD, high-food diet; Leu, leucine.

The effects of supplemental leucine on the leptin-signaling pathway in adipose tissues were shown in Fig. 4. The protein expression of ObR, JAK2, and STAT3 was significantly higher in the three leucine groups than in the HFD group (all *P*<0.01). However, SOCS3 expression was significantly lower in the HFD+1.5% Leu and HFD+4.5% Leu groups than in the HFD group (*P*<0.01). Notably, the expression of ObR, JAK2, and STAT3 was the highest, and the expression of SOCS3 was the lowest in HFD+1.5% Leu group (*P*<0.01, compared with the HFD+3.0% Leu and HFD+4.5% Leu groups).

## Discussion

In the present study, animals were exposed to HFD with or without leucine. Chronic leucine supplementation significantly decreased the circulating leptin level and increased the hypothalamic ObR. Accordingly, the protein expression of ObR, JAK2, and STAT3 increased, whereas that of SOCS3 decreased in adipose tissues.

Most animal studies that investigated the effect of leucine supplementation observed the weight/fat loss in diet-induced obese rats. Zhang found that leucine supplementation in drinking water can reduce the weight gain of HFD-fed mice by up to 32% (8). However, subsequent animal studies did not support the beneficial effect of leucine on body or adipose weight (10, 13, 15–17), which is consistent with our current result. Li even demonstrated that leucine supplementation in HFD increases body weight and perirenal white adipose tissue without altering calorie intake (18). Differences in animal trait, animal age, modeling method, supplemental timing, dosage, and intervention duration may be partially responsible for the divergent results in weight/fat loss.

At present, convincing evidence suggests that leucine benefits protein synthesis and improves insulin resistance and insulin sensitivity mainly through the mammalian target of rapamycin (mTOR) pathway (8, 11, 14, 18, 19). However, whether or not leucine supplementation increases leptin sensitivity in obese subjects with elevated leptin remains unclear. Several studies failed to observe the effect of leucine supplementation on circulating leptin level in obese animals (10, 15, 18). However, our current study demonstrated that leucine supplementation significantly lowers leptin level. This effect was not ascribed to the weight or adiposity loss related to the similarities of these indices among the groups in the present study.

To investigate leptin sensitivity, we further determined the ObR in the central nervous system and peripheral tissues. Result showed that leucine supplementation significantly increased the ObR in the hypothalamus and adipose tissues. However, owing to sample limitations, in our present study we were unable to measure the circulating level of soluble ObR, which has been hypothesized to control the availability of leptin to bind to ObR (20). Until now, no study investigated the effect of leucine supplementation on circulating soluble ObR. Thus, further studies are required to explore how leucine supplementation will affect circulating soluble ObR level and to what extent this will contribute to leucine's effects on other organs. On the other hand, Mao demonstrated that dietary leucine supplementation for 14 days significantly stimulates both the mRNA and protein expression of ObR in the skeletal muscles of mice (13). An *in vitro* experiment confirmed that the ObR expression is elevated in mouse C2C12 myotubes cultured with leucine for 2 h (13, 14). Binder directly observed the relationship between leucine supplementation and leptin action in previously obese mice. After 15 weeks of leucine supplementation, intraperitoneal injection with leptin significantly decreased food intake; however, this treatment did not affect food intake in the control group. Thus, they concluded that chronic leucine supplementation increases sensitivity to the anorectic action of leptin (10). In the current study, the improvement of leptin sensitivity by leucine supplementation was reflected in the findings of decreased circulating leptin level and increased ObR expression.

It is well known that ObR regulates the JAK2/STAT3 pathway, and ObR-induced STAT3 activation is essential for leptin regulation of energy balance (5, 21). To the best of our knowledge, our study is the first to investigate the effect of chronic leucine supplementation on the JAK2/STAT3 pathway. We found that leucine simultaneously supplemented with HFD increased the expression of JAK2 and STAT3, which is activated by ObR, in adipose tissues. In an acute intervention study, healthy volunteers who received leucine for 5 h showed an increase in the phosphorylation states of STAT3 in the duodenal mucosa; however, the mTOR pathway was unaffected (22). SOCS3, a negative feedback loop of the leptin-signaling pathway, can depress the JAK2/STAT3 pathway by binding to phosphorylated JAK (23). Inactivated SOCS3 in ObR-expressing cells increases the leptin sensitivity of HFD-exposed mice (24). Thus, the decreased SOCS3 expression in the present study partly explained the improvement of leptin sensitivity by leucine supplementation.

We did not observe a dose–response relationship, and even a relatively low supplementation of leucine (HFD+1.5% Leu group) showed a significant effect among the three leucine groups. Our result was similar to that of Li, who found that 1.5% leucine significantly affects the insulin sensitivity of rats on HFD (18). Leucine shares intestinal transporters with two other BCAAs (isoleucine and valine) and also participates in the intestinal absorption and cellular uptake of other amino acids, such as lysine, arginine, and histidine (25). Thus, it is likely that the availability of these amino acids will be interfered by the supplementation dosages of leucine. This was supported by the findings from other research groups although inconsistent results existed. For example, Zhang reported that although chronic leucine supplementation increased plasma leucine concentrations by 30% in diet-induced obese rats, it decreased the overall plasma amino acid levels (8). In another study, plasma leucine concentration was not significantly altered by leucine supplementation in rats previously exposed to a HFD (15). Therefore, the appropriate amount of leucine should be considered in practice. The other interesting result is that significant differences among leucine groups were observed in ObR expression and JAK2/STAT3 pathway, but not on body weight. In other words, single gene expression change by leucine supplementation cannot completely regulate the downstream consequence because several signaling pathways orchestrate the diet-induced body weight change. Thus, the beneficial roles of relative gene expression in leucine should not be overestimated in our study.

In conclusion, the present study is the first to demonstrate that chronic leucine supplementation can improve HFD-induced leptin resistance by decreasing the circulating leptin level and increasing the ObR. This improvement was ascribed to leptin signaling in adipose tissues, which increased the expression of JAK2 and STAT3 but decreased that of SOCS3.
